# Supplementary material for: Tunneling Nanotubes between Cells Migrating in ECM Mimicking Fibrous Environments
Source: Cancers (Basel). 2022 Apr 14;14(8):1989. doi: 10.3390/cancers14081989 (PMC9030013; doi:10.3390/cancers14081989)
Supplement: Supplementary file 1 [file cancers-14-01989-s001.zip › cancers-1662948-supplementary/Supplementary Materials.pdf]

Supplementary Materials: The following are available online at www.mdpi.com/xxx/s1,

Video S1: Time-lapse microscopy movie of TNT formation between MSTO-211H cells grown on a flat substrate.

Video S2: Time-lapse microscopy movie of TNT formation between MSTO-211H cells grown on a crosshatch nanofiber scaffold.

Video S3: Time-lapse microscopy movie of TNT formation between MSTO-211H cells grown on an aligned nanofiber scaffold.

Video S4: Time-lapse microscopy movie of TNT formation and bending on crosshatch nanofiber scaffold.

Video S5: 3D rendering of MSTO-211H cells grown on crosshatch nanofiber scaffold showing TNT bending around scaffold. Nanofiber scaffold is in red, MSTO-211H are in Green.

Video S6: Time-lapse microscopy movie of cargo moving along TNT that extend from cells grown on an aligned nanofiber scaffold. Red arrows indicate cargo.

Video S7: Time-lapse microscopy movie of cargo moving along bent TNT that extend from cells grown on a crosshatch nanofiber scaffold. Red arrows indicate cargo.
